# A cross-sectional study determining prevalence and factors associated with ST-segment elevation myocardial infarction and non-ST segment elevation myocardial infarction in Iran: results from fasa registry on acute myocardial infarction (FaRMI)

**DOI:** 10.1186/s12889-024-18140-6

**Published:** 2024-03-06

**Authors:** Mehdi Sharafi, Azizallah Dehghan, Ali Mouseli, Hossein Fatemian, Leila Jamali, Sima Afrashteh, Mahsa Rostami Chijan, Zahra Mastaneh, Abdoljabbar Zakeri, Abdulhakim Alkamel

**Affiliations:** 1 Cardiovascular Research Center, Hormozgan University of Medical Sciences, Bandar Abbas, Iran; 2 Noncommunicable Diseases Research Center, Fasa University of Medical Sciences, Fasa, Iran; 3 Social Determinants in Health Promotion Research Center, Hormozgan Health Institute, Hormozgan University of Medical Sciences, Bandar Abbas, Iran; 4 Student Research Committee, Shiraz University of Medical Sciences, Shiraz, Iran; 5 Department of Cardiovascular Medicine, Shiraz University of Medical Sciences, Shiraz, Iran; 6 Department of Biostatistics and Epidemiology, Faculty of Health and Nutrition, Bushehr University of Medical Sciences, Bushehr, Iran; 7 Department of Persian Medicine, Fasa University of Medical Sciences, Fasa, Iran; 8 Department of Health Information Management and Technology, School of Allied Medical Sciences, Hormozgan University of Medical Sciences, Bandar Abbas, Iran; 9 Department of Community Medicine, Faculty of Medicine, Hormozgan University of Medical Sciences, Bandar Abbas, Iran

**Keywords:** Prevalence, ST-segment, Non-ST segment, Myocardial infarction

## Abstract

**Background:**

Acute myocardial infarction is still a leading cause of death worldwide, accounting for roughly three million deaths yearly. This study aimed to investigate the prevalence and factors associated with ST-Segment Elevation Myocardial Infarction and Non-ST Segment Elevation Myocardial Infarction in Iran.

**Methods:**

This cross-sectional study was conducted using the databases of the Fasa Registry on Acute Myocardial Infarction (FaRMI) and the Fasa Adult Cohort Study (FACS). chi-squared and one-way ANOVA tests were utilized to calculate the unadjusted associations between the study variables. A multivariate multinomial logistic regression model was also employed to determine the adjusted association of each independent variable with the risk of ST-elevation myocardial infarction (STEMI).

**Results:**

The prevalence of STEMI and non-STEMI was 31.60% and 11.80%, respectively. Multinomial logistic regression showed that older age, anemia, high WBC, and high creatinine levels were associated with higher odds of STEMI and non-STEMI compared to healthy individuals. In addition, based on the analysis being a woman(OR = 0.63,95%CI:0.51–0.78), anemia(OR = 0.67,95%CI:0.54–0.63)and hypertension (OR = 0.80,95%CI:0.65–0.97)decreased the likelihood of STEMI occurrence compared to non-STEMI, while high WBC(OR = 1.19,95%CI:1.15–1.23)increased the odds.

**Conclusion:**

In this study, significant predictors of MI risk included age, gender, anemia, lipid profile, inflammation, and renal function. Subsequent investigations ought to prioritize the comprehensive understanding of the underlying mechanisms that drive these connections and assess the effectiveness of specific interventions aimed at diminishing the occurrence of MI and improving patient outcomes.

**Supplementary Information:**

The online version contains supplementary material available at 10.1186/s12889-024-18140-6.

## Introduction

Acute myocardial infarction is still a leading cause of death worldwide, accounting for roughly three million deaths yearly [1]. It is estimated that 23.6 million people will die from cardiovascular diseases (CVDs) by 2030 [2]. Acute coronary syndrome is divided into subgroups of ST-elevation myocardial infarction (STEMI), ST-elevation myocardial infarction (NSTEMI), and unstable angina [3]. The main pathogenesis of acute coronary syndrome is disorder or erosion in an atherosclerotic plaque, triggering the thrombosis cascade and reducing coronary blood flow. While STEMI and NSTEMI are similar in pathogenesis, the treatment protocol and prognosis differ [4]. STEMI involves a transmural infarction due to a complete coronary artery occlusion, necessitating emergency measures such as a percutaneous intervention (PCI) or thrombolytic therapy. This is while NSTEMI features an incomplete blockage of the coronary arteries, usually with a collateral flow to the threatened arterial territory [5–7].

Although the short-term mortality after STEMI is well recognized, the difference in long-term mortality between STEMI and non-STEMI is still debated [8–10]. Various studies have identified and confirmed factors that predict early mortality, but there is no classification and confirmation regarding those that predict late mortality [10–14]. Predictors of increased short-term mortality include ventricular tachycardia, ST-segment deviations, high-degree atrioventricular (AV) block, and prolonged QRS duration. Meanwhile, the most important independent risk factors for long-term mortality include ST segment deviations and left bundle-branch block (LBBB) [8]. The risk prediction tool for all forms of acute coronary syndrome identifies nine variables that predict six-month mortality: older age, history of myocardial infarction, history of heart failure, increased pulse rate at presentation, decreased systolic blood pressure at presentation, elevated initial serum creatinine level, elevated initial serum cardiac biomarker levels, ST-segment depression on presenting electrocardiogram, and not having a percutaneous coronary intervention performed in the hospital [14]. There is no similar study in the Iranian population to determine the difference in risk factor prevalence between STEMI and non-STEMI. Furthermore, we have a control group to determine the prevalence of risk factors in the normal population and compare it with MI patients. This study aimed to examine the prevalence and factors associated with ST-Segment Elevation Myocardial Infarction and Non-ST Segment Elevation Myocardial Infarction in Iran.

## Methods

This cross-sectional study was conducted using the databases of the Fasa Registry on Acute Myocardial Infarction (FaRMI) and the Fasa Adult Cohort Study (FACS).

### Fasa registry on acute myocardial infarction (FaRMI)

The FaRMI is a population-based registry aiming to improve the management and outcomes of patients with acute myocardial infarction (MI) in Iran. By providing a detailed description of patients’ characteristics, exploring management patterns, and investigating determinants of poor outcomes, this registry aims to improve the quality of care and build a foundation for further research in this area. This registry belongs to the Cardiology Department and Noncommunicable Diseases (NCD) Research Center of Fasa University of Medical Sciences (FUMS). Demographic data such as residence, ethnicity, past medical history, and risk factors were collected from participants. The clinical course of the patients is also recorded, including presenting symptoms, symptoms onset time, admission Killip class, and management in the pre-hospital setting, during the hospital stay, and at discharge. This comprehensive database provides valuable insights into the characteristics of patients with STEMI and NSTEMI, documenting the practice patterns and outcomes of MI in the region [15].

### Fasa adult Cohort study (FACS)

The FACS is a prospective cohort study that began in 2015 with an initial population of 10,000 individuals aged 35–70 in the Shasheda and Qara-Balagh regions of Fasa, Fars, Iran. The primary objective of this study is to investigate the risk factors associated with cardiovascular diseases. To collect the necessary information, a comprehensive questionnaire is used, which includes demographic information, history of communicable and non-communicable diseases, socioeconomic status, anthropometric measurements, body composition, electrocardiogram (ECG), blood pressure, food frequency questionnaire (FFQ), and biological samples (blood, nails, and hair). Trained interviewers are responsible for completing the questionnaires, and a supervisor checks the collected information before they are placed online on the server of the cohort center. This information can then be used to develop effective prevention and treatment strategies for individuals at risk of developing cardiovascular diseases [16].

## Measurements

In the FaRMI, patients are divided into two groups based on the type of MI: STEMI and non-STEMI. The study records various risk factors for these two groups, including demographic information, smoking status, body mass index (BMI), waist circumference, blood sugar, white blood cell count (WBC), creatinine levels, triglyceride levels, and low-density lipoprotein (LDL), high-density lipoprotein (HDL), and total cholesterol levels. To ensure a proper comparison and draw accurate conclusions, we randomly selected a healthy control group without any history of diseases from the FACS. We compared the STEMI, non-STEMI, and control groups regarding the recorded risk factors.

### Statistical analysis

We divided the participants into three groups: healthy controls, STEMI, and non-STEMI. We used different statistical methods to compare the risk factors between these groups. The frequency and percentage are reported for qualitative variables, while the mean and standard deviation are reported for quantitative variables. The chi-square, one-way ANOVA, and post hoc Tukey tests were used to assess differences between groups. Multinomial logistic regression was used in univariate and multivariate analyses to investigate the relationship and compare the two groups of MI with the control group. The normal group was considered the reference group. First, the univariate model was implemented and the predictor variables with *P* < 0.25 were included in the multivariate model. The Adjusted Odds Ratio (AOR) was used to report the effect size of any relationships, considering a 95% confidence interval. Furthermore, univariate and multivariate logistic regression were used to investigate the relationship between independent variables and STEMI/non-STEMI. In this model, the univariate model was used first. Variables with a significance level of less than 0.25 were then included in the multivariate model to control for confounding factors. The adjusted OR with a 95% confidence interval was used to report the association’s size. A P-value less than 0.05 was considered significant.

## Results

A total of 6,055 participants with a mean age of 53.62 ± 13.41 years were included in this analysis. The prevalence of STEMI and non-STEMI was 31.60% and 11.80%, respectively. Most subjects were men (64.50%), and 89.30% of the study population were married. Tobacco use was detected in 34.50% and 10.0% of STEMI and non-STEMI patients, respectively (*P* < 0.001). In addition, anemia was diagnosed in 48.60 and 24.30% of patients, respectively (*p* < 0.001). The mean BMI and waist circumference (WC) were 24.76 ± 4.16 and 91.40 ± 10.45 among study participants (*P* < 0.001). Mean blood glucose was higher in STEMI and non-STEMI patients than in normal subjects (*P* < 0.001), as were mean cholesterol, LDL, and triglyceride levels (*P* < 0.001). Participants’ mean WBC and creatinine levels were 7.833 ± 3.10 and 1.09 ± 0.39, respectively (*P* < 0.001). (Table 1). Since the one-way ANOVA test was significant, a post hoc Tukey test was performed to determine differences between groups. The results are shown in Supplementary Table 1.

**Table 1:** Characteristics of study participants (*N* = 6055)

**Characteristic**		**Category**	**Total**	**Normal** **3429 (56.60%)**	**STEMI** **1911 (31.60%)**	**Non-STEMI** **715 (11.80%)**	*P*-value
				**No. (%)**	**No. (%)**	**No. (%)**	
Age (Mean ± SD^*^			53.62 ± 13.41	46.75 ± 9.07	61.86 ± 12.91	64.53 ± 12.50	**< 0.001**
Sex**		Male	3903(64.50)	2092 (53.60)	1374 (35. 02)	437 (11.20)	**< 0.001**
		Female	2152(35.50)	1337 (62.10)	537 (25.00)	278 (12.90)	
Marital status**		Married	5406(89.30)	3114 (57.60)	1691 (31.30)	601 (11.10)	**< 0.001**
		Single	649(10.70)	315 (48.50)	220 (33.90)	114 (17.60)	
Tobacco smoking**		Yes	2343(38.70)	1299 (55.40)	809 (34.50)	235 (10.00)	**< 0.001**
		No	3712(61.30)	2130 (57.40)	1102 (29.70)	480 (12.90)	
Hypertension		Yes	1192(19.70)	0(0.00)	811(68.00)	381(32.00)	**< 0.001**
		No	4856(80.20)	3429(70.60)	1096(22.60)	331(6.80)	
Anemia		Yes	766(12.70)	186 (24.30)	372 (48.60)	186 (24.30)	**< 0.001**
		No	5289(87.30)	3243 (61.30)	1539 (29.10)	507 (9.60)	
Body mass index^*^			24.76 ± 4.16	24.59 ± 4.62	24.89 ± 3.35	25.25 ± 3.68	**< 0.001**
Waist circumference^*^			91.40 ± 10.45	90.15 ± 11.17	92.75 ± 9.36	93.74 ± 8.60	**< 0.001**
Blood sugar^*^			119.88 ± 65.87	88.50 ± 17.30	163.22 ± 79.13	154.49 ± 87.42	**< 0.001**
Total cholesterol^*^			191.60 ± 49.22	181.81 ± 40.83	204.07 ± 55.79	205.21 ± 56.09	**< 0.001**
Triglycerides^*^			148.57 ± 79.03	134.52 ± 77.25	164.64 ± 79.43	172.98 ± 72.10	**< 0.001**
LDL Cholesterol^*^			125.68 ± 43.72	111.74 ± 29.61	141.82 ± 54.57	149.42 ± 42.84	**< 0.001**
HDL Cholesterol*			46.38 ± 15.72	49.88 ± 15.54	42.12 ± 16.31	41.00 ± 9.29	**< 0.001**
WBC^*^			7.83 ± 3.10	6.448 ± 1.69	10.11 ± 3.61	8.43 ± 3.05	**< 0.001**
Creatinine^*^			1.09 ± 0.39	0.99 ± 0.18	1.21 ± 0.38	1.28 ± 0.81	**< 0.001**

*ANOVA

**Chi-squared test

STEMI: ST-elevation myocardial infarction; NSTEMI: non-ST-elevation myocardial infarction

Bold values ​​indicate a p-value less than 0.05 and its significance

Table 2 shows the results of multinomial logistic regression to identify associations between independent factors and STEMI/non-STEMI. With increasing age, the odds of STEMI (OR: 1.12, 95% CI: 1.11–1.14) and non-STEMI (OR: 1.14, 95% CI: 1.12–1.15) significantly increased compared to normal subjects. The odds of STEMI was significantly lower in women than in men (OR: 0.53, 95% CI: 0.39–0.73). With elevated blood lipid profile values, the odds of STEMI and non-STEMI increased compared to normal people, although these changes were not much. Compared to normal participants, anemia was associated with increased odds of non-STEMI (OR: 5.05, 95% CI: 3.41–7.46) and STEMI (OR: 3.47, 95% CI: 2.38–5.06). The odds of STEMI (OR: 1.74, 95% CI: 1.65–1.84) and non-STEMI (OR: 1.46, 95% CI: 1.38–1.56) increased with a one unit increase in white blood cells (WBC) compared to the normal group. In addition, elevated creatinine levels were associated with increased odds of non-STEMI (OR: 6.10, %95 CI: 3.64–10.23) and STEMI (OR: 4.45, %95 CI: 2 0.68–7.38).

**Table 2:** Summary of the findings of multinomial logistic regression of association between myocardial infarction and some of its risk factors (*N* = 6055)

Variables	Categories	Normal OR (95%CI)	STEMI OR (95%CI)	Non-STEMI OR (95%CI)
Age		1	1.12 (1.11–1.14) *	1.14 (1.12–1.15) *
Sex	♦ Male	**1**	**1**	**1**
	Female	1	0.53 (0.39–0.73) *	0.87 (0.64–1.23)
Tobacco smoking	♦No	1	**1**	**1**
	Yes	1	0.92 (0.71–1.20)	0.96 (0.64–1.17)
Anemia	♦No	**1**	**1**	**1**
	Yes	1	3.47 (2.38–5.06) *	5.05 (3.41–7.46) *
Body mass index		1	1.01 (0.97–1.03) *	1.03 (1.03–1.07) *
Blood sugar		**1**	1.05 (1.04–1.06) *	1.05 (1.03–1.06) *
Total cholesterol		1	1.03 (1.01–1.05) *	1.03 (1.01–1.06) *
Triglycerides		1	1.01 (1.01–1.03) *	1.02 (1.01–1.04) *
Low-density lipoprotein cholesterol		1	1.02 (1.01–1.02) *	1.02 (1.01–1.02) *
High-density lipoprotein cholesterol		**1**	0.95 (0.94–0.96) *	0.94 (0.93–0.95) *
White blood cells		1	1.74 (1.65–1.84) *	1.46 (1.38–1.56) *
Creatinine		1	4.45 (2.68–7.38) *	6.10 (3.64–10.23) *

*P-value < 0.05; OR: odds ratio; CI: confidence interval. The normal group was considered the reference group for multinomial regression analyses. ♦ indicates the reference category for each covariate. STEMI: ST-elevation myocardial infarction; NSTEMI: non-ST-elevation myocardial infarction

Table 3 shows the result of logistic regression to identify the differences in risk factors between STEMI and non-STEMI. In order of importance: being a woman (OR = 0.63, 95% CI:0.51–0.78, *P* < 0.001), anemia (OR = 0.67, 95% CI:0.54-0 0.63, *P* < 0.001), and hypertension (OR = 0.80, 95% CI: 0.65–0.97, *P* = 0.026) significantly reduced the odds of STEMI occurrence compared to non-STEMI. According to this analysis, with an increase of 1 unit of WBC, the odds of STEMI occurrence increased by 19% (OR = 1.19, 95% CI: 1.15–1.23, *P* < 0.001).

**Table 3:** Unadjusted and adjusted associations between the study variables and ST-elevation myocardial infarction (STEMI) *N* = 2626

Variable	Category	Crude Odds Ratio (95% CI)	*p*-value	Adjusted Odds Ratio (95% CI)	*p*-value
Age		0.98 (0.97–0.99)	< 0.001	0.99 (0.98-1.00)	0.053
Sex	♦Male	1	-	1	-
	Female	0.61 (0.51–0.73)	< 0.001	0.63 (0.51–0.78)	**< 0.001**
Marital status**	♦Single	1	-	1	-
	Married	1.45 (1.14–1.86)	0.003	NA	-
Tobacco smoking	♦No	1	-	1	-
	Yes	1.49 (1.25–1.79)	< 0.001	NA	-
Anemia	♦No	1	-	1	-
	Yes	0.83 (0.72–0.95)	0.009	0.67 (0.54–0.83)	**< 0.001**
Hypertension	♦No	1	-	1	-
	Yes	0.64 (0.54–0.76)	< 0.001	0.80 (0.65–0.97)	**0.026**
Body mass index		0.97 (0.94–0.99)	0.018	0.97 (0.94-1.00)	0.092
Waist circumference		0.98 (0.97–0.99)	0.014	0.99 (0.97-1.00)	0.127
Blood sugar		0.98 (0.97–0.99)	0.014	1.01 (1.01–1.03)	**0.004**
Total cholesterol		0.99 (0.99-1.00)	0.645	NA	-
Triglycerides		0.99 (0.98–0.99)	0.017	0.99 (0.99-1.00)	0.152
Low-density lipoprotein cholesterol		0.99 (0.98–0.99)	< 0.001	0.99 (0.98–0.99)	**0.008**
High-density lipoprotein cholesterol		1.00 (1.00,1.01)	0.045	1.00 (0.99–1.01)	0.175
White blood cells		1.19 (1.16–1.23)	< 0.001	1.19 (1.15–1.23)	**< 0.001**
Creatinine		0.79 (0.68–0.92)	0.004	0.72 (0.60–0.87)	**0.001**

Bold values ​​indicate a p-value less than 0.05 and its significance

## Discussion

This study sought to delineate the intricate relationship between demographic, clinical, and biochemical variables in predicting the likelihood of a myocardial infarction (MI). The findings of the present study revealed that age, anemia, white blood cell count, and creatinine levels were linked with an elevated probability of experiencing both forms of MI. The association between the female gender, anemia, and hypertension was more pronounced with non-STEMI than with STEMI. In addition, there were significant associations between blood lipid profiles and metabolic indicators and the risk of myocardial infarction, indicating their intricate influence on cardiovascular well-being.

The findings of this study indicate that age is a notable factor in the progression of both STEMI and non-STEMI. This observation aligns with prior studies that have emphasized the gradual influence of age on cardiovascular well-being [17–19]. According to the findings of the Framingham Heart Study, the incidence of MI over ten years was 12.9 per 1,000 individuals among males aged 30 to 34, while among females aged 35 to 44, the incidence was 5.2 per 1,000 [20, 21]. According to a study done in Joinville, Brazil, the overall incidence of stroke increased significantly by 62% in subjects under 45 years old and by 29% in subjects under 55 years old, suggesting a rising trend in cardiovascular events in younger age groups. [22]. 

The incidence of acute MI in individuals under 40 appears to have been rising, according to epidemiological data [23, 24]. The risk factor profile of myocardial infarction (MI) differs in younger patients (≤ 40 years old) than in older patients [25]. Some notable differences are as follows: increased drug abuse, decreased hypertension, eccentric atherosclerotic plaques with inflammatory properties, obesity, smoking, and unhealthy lifestyle habits. [25]. In addition, a large proportion of young people with acute STEMI had traditional risk factors such as obesity, diabetes mellitus, hypertension, and smoking [26]. Very young MI patients had a similar risk of cardiovascular death and all-cause mortality as older patients over a median follow-up of 11.2 years [25]. Nevertheless, the pathophysiology of CHD in younger patients differs slightly from that in older patients. Young MI patients have a higher percentage of single-vessel coronary lesions and a higher likelihood of a right-dominant distribution of coronary artery lesions [23]. There is less collateral circulation and a lower degree of coronary artery stenosis than in older patients. Different populations have different prevalence rates of traditional risk factors for AMI, such as a positive family history, smoking, diabetes mellitus, hypertension, dyslipidemia, and a sedentary lifestyle [23, 27]. 

The likelihood of developing any form of MI positively correlates with advancing age, underscoring the significance of age as an unalterable risk factor. The heightened vulnerability to MI that occurs with age progression is related to several physiological alterations within the cardiovascular system. The incidence of MI rises in older adults due to various age-related variables, including arterial stiffening, atherosclerosis, diminished collateral circulation, cellular aging, and cumulative exposure to risk factors such as hypertension and high cholesterol. Moreover, chronic inflammation and a decline in regeneration capability also significantly exacerbate this heightened susceptibility. Although age is a significant risk factor, it is essential to recognize the complex interaction of these factors, emphasizing the necessity of proactive cardiovascular healthcare in all age cohorts to reduce the probability of experiencing an MI [28–31].

A notable gender disparity was observed in the present research, wherein women demonstrated a decreased likelihood of developing STEMI than men. The observed gender gap is consistent with previous research identifying a higher incidence rate of MI in males than females. Approximately 70% of MI instances are reported in males, who typically have an MI 7–10 years earlier than females [32, 33]; this demonstrates that women have a reduced risk of developing STEMI [34].

Men and women have different risks of experiencing ST-elevation myocardial infarction (STEMI), which is influenced by different factors. According to a Middle East study, women with acute STEMI were more likely to be older than men and to have diabetes mellitus, hypertension, and hyperlipidemia. In addition, the average time between the onset of symptoms and arrival in the emergency room was longer and the likelihood of receiving timely and effective care was lower. As a result, in this specific region, the mortality rate due to STEMI was almost twice as high in women as in men [35]. According to other Chinese studies, a significant proportion of STEMI patients without standard modifiable cardiovascular risk factors (SMuRFs) had higher mortality rates and were less likely to receive specific treatments than patients with SMuRFs [36, 37]. 

The results indicate that anemia is a notable risk factor for STEMI and non-STEMI; persons diagnosed with anemia demonstrated a significantly increased likelihood of experiencing an MI. The prevalence of anemia in patients with acute coronary syndrome (ACS) at admission varies between 10% and 43%, with a potential increase to 57% throughout their hospitalization due to the development of hospital-acquired anemia (HAA). Both admission anemia and hospital-acquired anemia (HAA) are associated with higher rates of short- and long-term mortality [38, 39]. However, their prognostic importance is influenced by distinct mechanisms. Anemia at baseline is frequently associated with prior medical issues requiring further evaluation and intervention. The relationship between high altitude acclimatization (HAA) and clinical features, medical treatments, and interventions is known to elicit cardiovascular changes that potentially exacerbate myocardial ischemia [38]. Anemia is a medical condition generally identified by a decrease in the blood’s ability to carry oxygen. This condition can worsen the imbalance between the demand and supply of oxygen in the heart muscle, increasing the likelihood of experiencing an MI [40]. Additional research is necessary to clarify the underlying mechanisms that connect anemia with the risk of MI.

The present research also highlights the significance of lipid profiles in relation to MI risk; heightened total cholesterol, LDL cholesterol, and triglyceride concentrations correlated with an increased likelihood of experiencing STEMI/non-STEMI. These findings are consistent with previous research demonstrating an elevated dyslipidemia prevalence in patients with ACS, regardless of their obesity status [41, 42]. These results emphasize the crucial role of lipid management in cardiovascular disease prevention and management.

We found that the WBC count and creatinine level were significant factors in assessing the risk of MI, indicating inflammation and renal function, respectively. Increments in these parameters correlated with a greater likelihood of experiencing both types of MI. previous studies indicate that WBC count could be a predictor for mortality in peripheral artery diseases [43]. The decrease in blood flow within the epicardium and impaired myocardial perfusion, along with increased thromboresistance, have been associated with a heightened occurrence of new cases of congestive heart failure and mortality [44]. Moreover, it should be noted that WBC serves as a significant prognostic indicator for long-term mortality in patients with both non-STEMI and STEMI [45]. Plasma creatinine was also described as an important factor in previous studies, An elevated level of serum creatinine within the established normal range serves as an indicator for heightened susceptibility to cerebrovascular disease in individuals who are both normotensive and hypertensive [46]. Myocardial infarction, ischemic heart disease, and premature death were linked to moderately elevated plasma creatinine levels. An association was not found for low estimated glomerular filtration rate (eGFR) [47]. There exists a notable correlation between serum creatinine levels and coronary artery disease (CAD), although it should be noted that this correlation is not independent [48]. The findings above underscore the complex interconnections between inflammation, renal function, and cardiovascular well-being, indicating that therapies aimed at addressing these variables may have the potential to mitigate the risk of MI.

Notably, although tobacco use did not emerge as a significant predictor in our adjusted analysis, its frequency was considerable among patients diagnosed with both forms of MI. Efforts aimed at smoking cessation and public health programs specifically addressing tobacco use remain crucial in the ongoing endeavor to mitigate the burden of cardiovascular disease [49].

The study’s notable qualities are rooted in thoroughly examining a sizable and varied group, yielding valuable observations regarding the complex aspects of MI risk. Nevertheless, it is crucial to take into account several constraints. However, some limitations must be acknowledged. The cross-sectional design prevented causation, so future studies must confirm the observed relationships. Heavy use of self-reported data may introduce recall bias and inaccuracies, especially for lifestyle factors like smoking. The study is limited to Fasa, Iran, and may not be representative of other populations. Omitting variables like genetic predisposition or environmental factors may also reduce analysis comprehensiveness. Finally, the study’s robust statistical methods ignore potential confounding factors that could affect observed associations.

## Conclusions

Research sheds light on the complex network of risk variables contributing to STEMI and NSTEMI. Significant predictors of MI risk included age, gender, anemia, lipid profile, inflammation, and renal function. These findings highlight the need to conduct thorough risk assessments and implement customized interventions to alleviate cardiovascular disease’s burden on the community. Subsequent investigations ought to prioritize the comprehensive understanding of the underlying mechanisms that drive these connections and assess the effectiveness of specific interventions aimed at diminishing the occurrence of MI and improving patient outcomes.

### Electronic supplementary material

Below is the link to the electronic supplementary material.

Supplementary Material 1: Multiple/Post Hoc group comparisons of demographic and clinical factors in myocardial infarction

## Data Availability

The data of this study is not publicly available due to its being the intellectual property of Fasa University of Medical Sciences but is available from the corresponding author on a reasonable request.
