# Supplementary material for: A cross-sectional study determining prevalence and factors associated with ST-segment elevation myocardial infarction and non-ST segment elevation myocardial infarction in Iran: results from fasa registry on acute myocardial infarction (FaRMI)
Source: BMC Public Health. 2024 Mar 6;24:728. doi: 10.1186/s12889-024-18140-6 (PMC10918930; doi:10.1186/s12889-024-18140-6)
Supplement: Supplementary file 1 — Supplementary Material 1: Multiple/Post Hoc group comparisons of demographic and clinical factors in myocardial infarction [file 12889_2024_18140_MOESM1_ESM.docx]

Table S1: Multiple/Post Hoc group comparisons of demographic and clinical factors in myocardial infarction

| **Dependent Variable** | | **(I) groups** | **(J) groups** | **Mean Difference (I-J)** | **Std. Error** | **Sig.** | **95% Confidence Interval** | |
| --- | --- | --- | --- | --- | --- | --- | --- | --- |
|  |  |  |  |  |  |  | **Lower Bound** | **Upper Bound** |
| **Age** | Tukey | STEMI | Non-STEMI | -2.670^*^ | 0.47 | <0.001 | -3.78 | -1.55 |
|  |  |  | Normal | 15.108^*^ | 0.31 | <0.001 | 14.38 | 15.83 |
|  |  | Non-STEMI | STEMI | 2.670^*^ | 0.47 | <0.001 | 1.55 | 3.78 |
|  |  |  | Normal | 17.778^*^ | 0.44 | <0.001 | 16.73 | 18.82 |
|  |  | Normal | STEMI | -15.108^*^ | 0.31 | <0.001 | 15.83 | -14.38 |
|  |  |  | Non-STEMI | -17.778^*^ | 0.44 | <0.001 | -18.82 | -16.73 |
| **Body mass index** | Tukey | STEMI | Non-STEMI | -0.360 | 0.18 | 0.118 | -0.78 | 0.06 |
|  |  |  | Normal | -0.303^*^ | 0.11 | 0.029 | 0.02 | 0.58 |
|  |  | Non-STEMI | STEMI | 0.360 | 0.18 | 0.118 | -0.06 | 0.78 |
|  |  |  | Normal | 0.663^*^ | 0.17 | <0.001 | 0.26 | 1.06 |
|  |  | Normal | STEMI | -0.303^*^ | 0.11 | 0.029 | -0.58 | -0.02 |
|  |  |  | Non-STEMI | -0.663^*^ | 0.17 | <0.001 | -1.06 | -0.26 |
| **Waist circumference** | Tukey | STEMI | Non-STEMI | -0.990 | 0.45 | 0.074 | -2.05 | 0.07 |
|  |  |  | Normal | 2.604^*^ | 0.29 | <0.001 | 1.91 | 3.29 |
|  |  | Non-STEMI | STEMI | 0.990 | 0.45 | 0.074 | -0.07 | 2.05 |
|  |  |  | Normal | 3.594 | 0.42 | <0.001 | 2.59 | 4.59 |
|  |  | Normal | STEMI | -2.604 | 0.29 | <0.001 | -3.29 | -1.91 |
|  |  |  | Non-STEMI | -3.594 | 0.42 | <0.001 | -4.59 | -2.59 |
| **Blood sugar** | Tukey | STEMI | Non-STEMI | 8.713^*^ | 2.42 | 0.001 | 3.05 | 14.40 |
|  |  |  | Normal | 74.423^*^ | 1.57 | <0.001 | 71.02 | 78.41 |
|  |  | Non-STEMI | STEMI | -8.731^*^ | 2.42 | 0.001 | -14.40 | -3.05 |
|  |  |  | Normal | 65.992^*^ | 2.26 | <0.001 | 60.67 | 71.31 |
|  |  | Normal | STEMI | -74.723^*^ | 1.57 | <0.001 | -78.41 | -71.02 |
|  |  |  | Non-STEMI | -65.992^*^ | 2.26 | <0.001 | -71.31 | -60.67 |
| **Total cholesterol** | Tukey | STEMI | Non-STEMI | -1.138 | 2.10 | 0.851 | -6.06 | 3.78 |
|  |  |  | Normal | 22.259^*^ | 1.36 | <0.001 | 19.05 | 25.46 |
|  |  | Non-STEMI | STEMI | 1.138 | 2.10 | 0.851 | -3.78 | 6.06 |
|  |  |  | Normal | 23.398^*^ | 1.97 | <0.001 | 18.77 | 28.01 |
|  |  | Normal | STEMI | -22.259^*^ | 1.36 | <0.001 | -25.46 | -19.05 |
|  |  |  | Non-STEMI | -23.398^*^ | 1.97 | <0.001 | -28.01 | -18.77 |
| **Triglycerides** | Tukey | STEMI | Non-STEMI | -8.338^*^ | 3.39 | 0.037 | -16.28 | -0.38 |
|  |  |  | Normal | 30.120^*^ | 2.20 | <0.001 | 24.94 | 35.29 |
|  |  | Non-STEMI | STEMI | 8.338^*^ | 3.39 | 0.037 | 0.38 | 16.28 |
|  |  |  | Normal | 38.495^*^ | 3.18 | <0.001 | 31.00 | 45.91 |
|  |  | Normal | STEMI | -30.120^*^ | 2.20 | <0.001 | -35.29 | -24.94 |
|  |  |  | Non-STEMI | -38.459^*^ | 3.18 | <0.001 | -45.91 | -31.00 |
| **LDL Cholesterol** | Tukey | STEMI | Non-STEMI | -7.595^*^ | 1.78 | <0.001 | -11.77 | -3.41 |
|  |  |  | Normal | 30.083^*^ | 1.16 | <0.001 | 27.36 | 32.80 |
|  |  | Non-STEMI | STEMI | 7.595* | 1.78 | <0.001 | 3.41 | 11.77 |
|  |  |  | Normal | 37.678^*^ | 1.67 | <0.001 | 33.75 | 41.59 |
|  |  | Normal | STEMI | -30.083^*^ | 1.16 | <0.001 | -32.80 | -27.36 |
|  |  |  | Non-STEMI | -37.678^*^ | 1.67 | <0.001 | -41.59 | -33.75 |
| **HDL Cholesterol** | Tukey | STEMI | Non-STEMI | 1.111 | 0.666 | 0.218 | -0.45 | 2.67 |
|  |  |  | Normal | -7.767^*^ | 0.432 | <0.001 | -8.78 | -6.74 |
|  |  | Non-STEMI | STEMI | -1.111 | 0.666 | 0.218 | -2.67 | 0.45 |
|  |  |  | Normal | -8.878^*^ | 0.625 | <0.001 | -10.34 | -7.41 |
|  |  | Normal | STEMI | 7.767^*^ | 0.434 | <0.001 | 6.74 | 8.78 |
|  |  |  | Non-STEMI | 8.878^*^ | 0.625 | <0.001 | 7.41 | 10.34 |
| **WBC** | Tukey | STEMI | Non-STEMI | 1.682^*^ | 0.11 | <0.001 | 1.41 | 1.95 |
|  |  |  | Normal | 3.677^*^ | 0.07 | <0.001 | 3.50 | 3.85 |
|  |  | Non-STEMI | STEMI | -1.682^*^ | 0.11 | <0.001 | -1.95 | -1.41 |
|  |  |  | Normal | 1.994^*^ | 0.10 | <0.001 | 1.74 | 2.24 |
|  |  | Normal | STEMI | -3.677^*^ | 0.07 | <0.001 | -3.85 | -3.50 |
|  |  |  | Non-STEMI | -1.994^*^ | 0.10 | <0.001 | -2.24 | -1.74 |
| **Creatinine** | Tukey | STEMI | Non-STEMI | -0.073^*^ | -0.073^*^ | <0.001 | -0.112 | -0.034 |
|  |  |  | Normal | 0.212^*^ | 0.212^*^ | <0.001 | 0.187 | 0.237 |
|  |  | Non-STEMI | STEMI | 0.073^*^ | 0.073^*^ | <0.001 | 0.034 | 0.112 |
|  |  |  | Normal | 0.285^*^ | 0.285^*^ | <0.001 | 0.249 | 0.322 |
|  |  | Normal | STEMI | -0.212^*^ | -0.212^*^ | <0.001 | -0.237 | -0.187 |
|  |  |  | Non-STEMI | -0.285^*^ | -0.285^*^ | <0.001 | -0.322 | -0.249 |
| *. The mean difference is significant at the 0.05 level. | | | | | | | | |
